# The PARPscore system using poly (ADP-ribose) polymerase (PARP) family features and tumor immune microenvironment in glioma

**DOI:** 10.1007/s12672-024-01734-2

**Published:** 2024-12-26

**Authors:** Cheng Zhang, Juan Feng, Xia Zhou, Jie Zhang, Chuming Tao, Hongwei Zhou

**Affiliations:** 1 Department of Neurosurgery, Zigong Third People’s Hospital, Zigong, 643020 Sichuan China; 2 Operating Room, Zigong Third People’s Hospital, Zigong, 643020 Sichuan China; 3 Department of Neurology, Fushun People’s Hospital, Fushun, 643200 Sichuan China; 4 Department of Science and Education, Zigong Third People’s Hospital, Zigong, 643020 Sichuan China; 5 Department of Cerebrovascular Disease, Suining First People’s Hospital, No. 2 Wentao Road, High-Tech Zone, Suining, 629000 Sichuan China

**Keywords:** PARPs, Tumor microenvironment, Glioma, Immunotherapy, Mutation burden

## Abstract

The immune response plays a pivotal role in tumor progression and therapy. However, the influence of protein PAR polymerases (PARPs) modifications on cell infiltration within the tumor microenvironment (TME) remains insufficiently understood. In this study, the Clinical and RNA sequencing data we performed a comprehensive analysis of PARPs modification patterns, exploring their associations with TME cell infiltration were acquired from the Cancer Genome Atlas (TCGA) and Chinese Glioma Genome Atlas (CGGA) database. To quantify PARPs modification in individual tumors, we developed a novel metric, the PARPscore, derived using principal component analysis. Our findings revealed three distinct PARPs modification patterns, each correlated with unique TME infiltration characteristics and tumor immunophenotypes. These patterns demonstrated predictive value for various clinical parameters, including inflammation stage, tumor subtypes, TME matrix activity, genetic variations, and patient prognosis. Notably, the high PARPscore subtype exhibited features of stromal activation and reduced immune infiltration, indicative of a non-inflamed, immune-excluded TME phenotype, and was associated with poorer survival outcomes. Conversely, lower PARPscore subtypes corresponded to substantial therapeutic benefits and improved outcomes in two independent immunotherapy cohorts. This study underscores the critical role of PARPs modification in shaping the diverse and dynamic TME. By delineating tumor-specific PARPs modification patterns, we provide valuable insights into TME complexity and its implications for immunotherapy.

**Supplementary Information:**

The online version contains supplementary material available at 10.1007/s12672-024-01734-2.

## Introduction

Adenosine diphosphate (ADP)-ribosylation (ADPr) is a post-translational modification involved in key cellular processes, including DNA damage repair, cell proliferation and differentiation, metabolism, stress responses, and immune regulation. The poly (ADP-ribose) polymerase (PARP) family acts as ADP-ribose “writers,” covalently attaching ADP-ribose units to substrate proteins. PARP-mediated modifications have been implicated in various human diseases, such as diabetes, neurological disorders, cardiovascular conditions, and cancer [1–4]. Numerous PARP inhibitors have been developed and are used to treat cancers, including breast and ovarian cancers [5]. Of particular interest, PARPs play a critical role in regulating innate immune responses, including anti-tumor and inflammatory processes [6]. This will help us further investigate the modified effects of PARPS, as well as its novel mechanisms in anti-tumor.

Glioma is the most common primary malignant brain tumors in humans, accounting for 81% of central nervous system (CNS) malignancies [7, 8]. Existing treatment modalities include surgery and chemoradiotherapy, but the prognosis improvement in patients with glioma are very limited, the median survival time for glioblastoma (GBM) patients is merely approximately 14 months [9, 10]. However, modulating the modification of PARPs has been reported to have significant effects in the treatment of gliomas, and the use of PARP inhibitors can also enhance the sensitivity of tumor cells to chemoradiotherapy [11]. Therefore, the analysis aimed at studying the immune mechanisms of PARP regulating in gliomas is particularly important. Moreover, some emerging immunotherapies are effective in the treatment of tumors. Studies have shown that blocking therapy for immune checkpoints can improve survival for multiple tumor types, including gliomas [9, 12–14]. Research has shown a link between PARPs and immunotherapy for tumors [15–17]. For example, Seyedin et al. [18] found that PARP inhibition can enhance tumor antigen presentation and responsiveness to anti-PD-1 therapy in colorectal tumor models. PARP inhibition elicits sting-dependent antitumor immunity in brca1-deficient ovarian cancer [19]. However, to date, the effects of using PARP modulators on the tumor microenvironment and the immune system in immunotherapies targeting PARP remain unknown.

In this study, we collected and combined datasets from two CGGA gliomas and identified three patterns of PARPs through consensus clustering. And the TME characteristics in these three modes were highly consistent with the immuno-exclusion phenotype, immunoingenic phenotype, and immune desert phenotype, respectively. These findings suggest a pivotal role of PARPs in modifying the individual tumor microenvironment. Simultaneously, we devised a scoring system to quantify each patient's PARPs pattern.

## Methods

### Dataset collection

The flowchart illustrating the study methodology is presented in Fig. S1A. 3 glioma cohorts (CGGA1, CGGA2, and TCGA) were gathered in this study for further analysis. The CGGA datasets were downloaded from the Chinese Glioma Genome Atlas (http://www.cgga.org.cn/index.jsp), and TCGA dataset was downloaded from the Genomic Data Commons (GDC, https://portal.gdc.cancer.gov/). The clinical information of all datasets is counted in supplementary table S1. Somatic mutation (SNP) downloaded from the University of California Santa Cruz (UCSC) Xena browser.

### Validation of bioinformatics results was carried out using reverse transcription quantitative polymerase chain reaction (RT-qPCR)

We collected normal brain tissue (NBT) as well as glioma tissues, including five NBT samples, five low-grade glioma (LGG) tissues, and five samples of GBM from the Department of Neurosurgery, Zigong Third People’s Hospital, during the period between November 2022 and December 2022. The reverse transcription quantitative polymerase chain reaction (RT-qPCR) assay was performed using a LightCycler® 480 real-time PCR system following the manufacturer's guidelines. Gene expression levels were determined using the 2-ΔΔCt method. The primer sequences for the genes can be found in supplementary table S2. All patients provided written informed consent after receiving detailed information about the study, including its purpose and publication of findings. The study adhered to the principles of the Helsinki Declaration and received approval from the Ethics Committee of Zigong Third People’s Hospital (2023-009).

### Consensus clustering for 17 PARPs

Seventeen PARP genes derived from the review by Kim et al. [20], which summarized the number as well as function of PARPs, it contains 13 poly (ADP-ribose) (PARP1, PARP2, PARP3, PARP4, PARP6, PARP8, PARP9, PARP10, PARP11, PARP12, PARP14, PARP15, PARP16), 2 tankyrase (TNKS, TNKS2) 1 zinc finger (ZC3HAV1) and 1 TCDD (TIPARP). Expression matrix of 17 PARPs were extracted from the two combined CGGA datasets to identify different patterns of PARPs modification mediated by PARPs. Based on the expression profiles of 17 PARPs, we utilized the "ConsensusClusterPlus" R package to identify distinct PARP modification patterns [21]. We apply "limma" R packet to identify differentially expressed genes (DEGs) between different patterns [22]. The adjusted P-value in DEGs < 0.001 is considered a gene with significant differences.

### Gene set variation analysis (GSVA) and functional annotation

The R package "GSVA" is used to study changes in various biological processes under the PARPs modification patterns [23]. The Hallmarker gene set is used to define biological characteristics [24].

### Estimation of immune cell infiltration using single-sample gene set enrichment analysis (ssGSEA) and deconvolution algorithm

SsGSEA was used to identify the relative abundance of 28 immune cell types in gliomas [25, 26]. The relative abundance of each immune cell type was quantified by an enrichment score through ssGSEA analysis, normalized to a unity distribution ranging from 0 to 1. The biosimilarity of infiltrating immune cells was estimated using multidimensional scaling (MDS) and a Gaussian fitting model.

### Generation of PARPs signature

We created a scoring system to evaluate the PARPs modification patterns of glioma patients, which we call PARPscore. The construction steps for PARPs signature are as follows: First, the DEGs identified from different ADP clusters are normalized in samples from all CGGA databases, and the overlapping genes are extracted. We employed a univariate Cox regression model for prognostic analysis of the selected genes. Subsequently, a consensus clustering method was applied to categorize all samples into distinct groups. Following this, we conducted principal component analysis (PCA) to identify PARPs-related genetic signatures, reducing redundancy and complexity in our analysis. Both principal components 1 and 2 were chosen as signature scores. We then adopted a formula similar to previous studies to define the PARPscore [27, 28]:

$$\text{PARPscore}={\sum }\left({PC1}_{i}+P{C2}_{i}\right)$$

where i is the expression of PARPs.

### Collect immunotherapy datasets and clinical information

We collected two immunotherapy cohorts from the geo database: advanced urinary urothelial carcinoma, anti-PD-L1 antibody atezolizumab intervention (IMvigor210 cohort) [29], bevacizumab combination treatment and after bevacizumab combination treatment in both responding and non-responding recurrent glioblastoma (GSE79671) [30], and obtained relevant clinical information files.

### Statistical analysis

We calculated correlation coefficients between TME-infiltrating immune cells and PARPs expression using Spearman and distance correlation analyses. For comparison of differences among three or more groups, we utilized one-way analysis of variance and Kruskal–Wallis tests [31]. We utilized the PARPscore to determine the optimal cutoff using the "survminer" R package. The "surv-cutpoint" function repeatedly tested potential cutoffs, identifying the maximum rank statistic. We dichotomized the PARPscore based on this cutoff, dividing patients into high and low PARPscore subgroups to mitigate calculated batch effects. Prognostic analysis involved generating Kaplan–Meier survival curves and employing the log-rank test for significance determination. Univariate Cox regression calculated hazard ratios (HR) for PARPs and PARP phenotype-related genes. Independent prognostic factors were identified using multivariate Cox regression models. In the TCGA cohort, the "maftools" R package's waterfall function illustrated mutations in patients with high and low PARPscore subtypes. Furthermore, we used the "RCircos" R package to map copy number variations for the 23 pairs of 17 PARPs across chromosomes [32]. All p-values for statistical tests were two-sided, and a significance level of p < 0.05 was used to determine statistical significance. Data processing was performed using R version 4.2.2 software.

## Results

### Landscape of genetic variation of PARPs in glioma

**Fig. 1:**
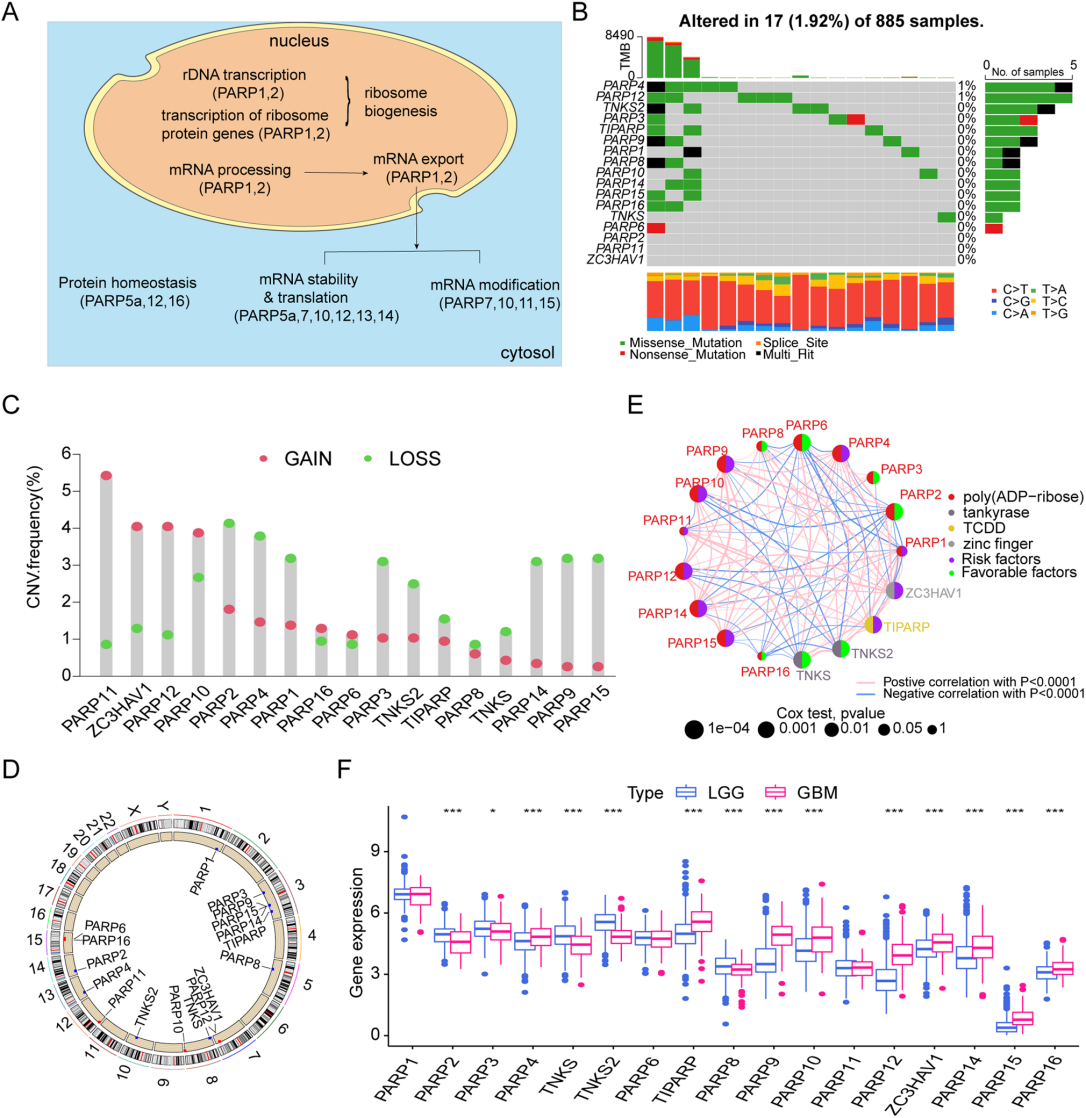
Genetic Variation Landscape of PARPs in Glioma. **A** Functional roles of 17 PARPs in cellular biology. **B** Mutation frequency of 17 PARPs in the TCGA cohort. **C** Copy number variation (CNV) frequency of PARPs in the TCGA cohort. **D** Chromosomal location of CNV alterations of PARPs across 23 chromosomes using the TCGA cohort. **E** Protein–Protein Interaction (PPI) network among PARPs. **F** Box plot illustrating the expression levels of the 17 PARPs between Lower Grade Glioma (LGG) and Glioblastoma (GBM) in the TCGA cohort. *p < 0.05, ***p < 0.001

After considering a systematic review of published articles about PARPs. A total of 17 PARPs were collected. The PARPs including 13 poly(ADP-ribose) (PARP1, PARP2, PARP3, PARP4, PARP6, PARP8, PARP9, PARP10, PARP11, PARP12, PARP14, PARP15, and PARP16), 2 tankyrase (TNKS, TNKS2) 1 zinc finger (ZC3HAV1) and 1 TCDD (TIPARP). The process of these PARPs involved in many biological processes, such as mRNA modification, transcription of ribosome protein genes, and protein homeostasis (Fig. 1A). In the glioma genome, the mutation rate for all PARPs is relatively low. Of the 885 (1.92%) samples, a total of 17 samples had genetic changes in PARPs, including missense mutations and splicing site mutations. Among them, the highest mutation frequency is PARP4, the followed are PARP12 and TNKS2 (Fig. 1B). The copy number variation (CNV) of PARP11, ZC3HAV1, and PARP12 is mainly manifested as copy amplification; and TIPARP, PARP8, and TNKS monetize missing copy numbers (Fig. 1C). The location of CNV alteration of PARPs on chromosomes was shown in Fig. 1D. The protein–protein interaction (PPI) network performed by string demonstrates a wide range of interactions between these PARPs (Fig. 1E, S1B). Additionally, the analysis of PARPs expression differences between LGG and GBM revealed significant variations in most PARPs across different grades of gliomas, underscoring their potentially significant role in the malignant progression of gliomas (Fig. 1F). Based on the findings in Fig. 1F, we identified genes displaying significant expression differences between LGG and GBM. In addition, we tested the RNA expression levels of these genes in gliomas of different grades by qRT-PCR assays. Fig. S2 illustrates that 14 PARPs exhibited differential expression patterns across normal brain tissues, LGG, and GBM tissues (Fig. S2A-N). These findings revealed significant differences in epigenetic and gene expression of PARPs between LGG and GBM.

### The modification patterns of 17 PARPs in glioma

**Fig. 2:**
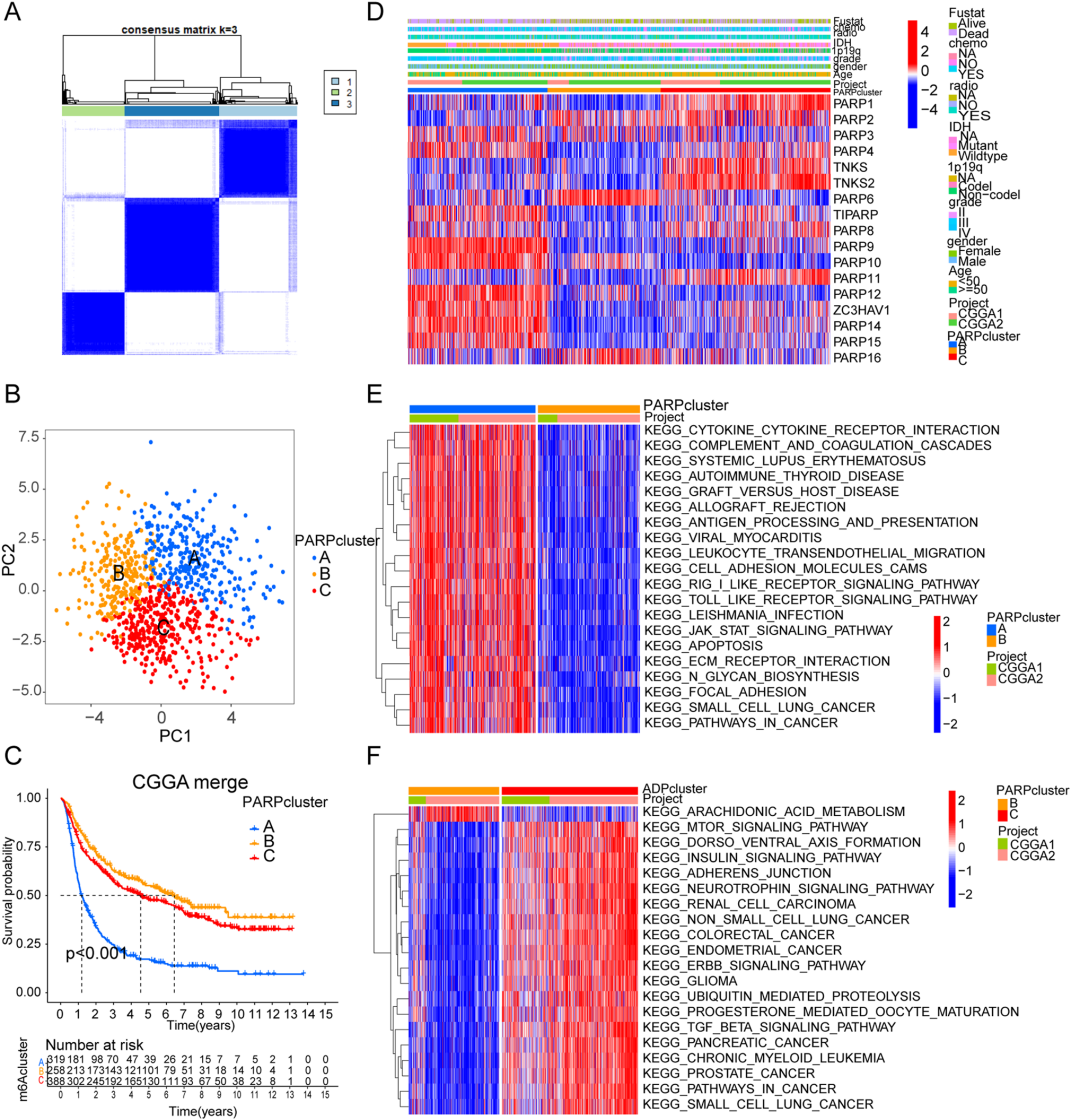
PARPs Modification Patterns and Relevant Biological Pathways. **A** By consensus clustering, all glioma patients were divided into 3 clusters. **B** Principal Component Analysis (PCA) was employed to validate the presence of three discernible patterns, determined by the expression profiles of the 17 PARPs across a total of 2228 glioma samples. **C** Survival curves of glioma samples categorized into the three PARPs modification patterns within the combined glioma cohorts. **D** Heatmap illustrating consensus clustering based on 17 PARPs. **E**, **F** GSVA analysis displaying the enrichment of gene sets in the three patterns

The two CGGA datasets, CGGA1 and CGGA2 (supplementary table S1), were integrated into a single cohort. Relevant survival data and clinical information were compiled. Prognostic assessment of 17 PARPs in glioma patients was conducted using the univariate Cox regression model (Fig. S1C). Utilizing the expression of the 17 PARPs, PCA analysis distinguished three PARP modification patterns. The results demonstrated the effectiveness of these patterns in distinguishing the samples (Fig. 2A and B). Survival analysis indicated a notable survival advantage associated with the PARP cluster B modification pattern, whereas cluster A exhibited the worst prognosis (Fig. 2C). Using the “ConsensusClusterPlus” R package, patients were categorized into distinct PARP modification patterns based on the expression profiles of 17 PARPs. Unsupervised clustering identified three unique modification patterns: 319 cases in pattern A, 258 in pattern B, and 388 in pattern C (Fig. 2A and Fig. S3A–H). The “ConsensusClusterPlus” R package was utilized to classify patients based on qualitatively different PARP modification patterns derived from the expression of these 17 PARPs. Unsupervised clustering revealed three distinct modification patterns: 319 cases in pattern A, 258 cases in pattern B, and 388 cases in pattern C (Fig. 2A and Fig. S3A–H). These patterns were termed as PARP clusters A–C, respectively (Fig. 2D and supplementary table S3).

### Characteristics of tumor microenvironment cell infiltration in different modification patterns

**Fig. 3:**
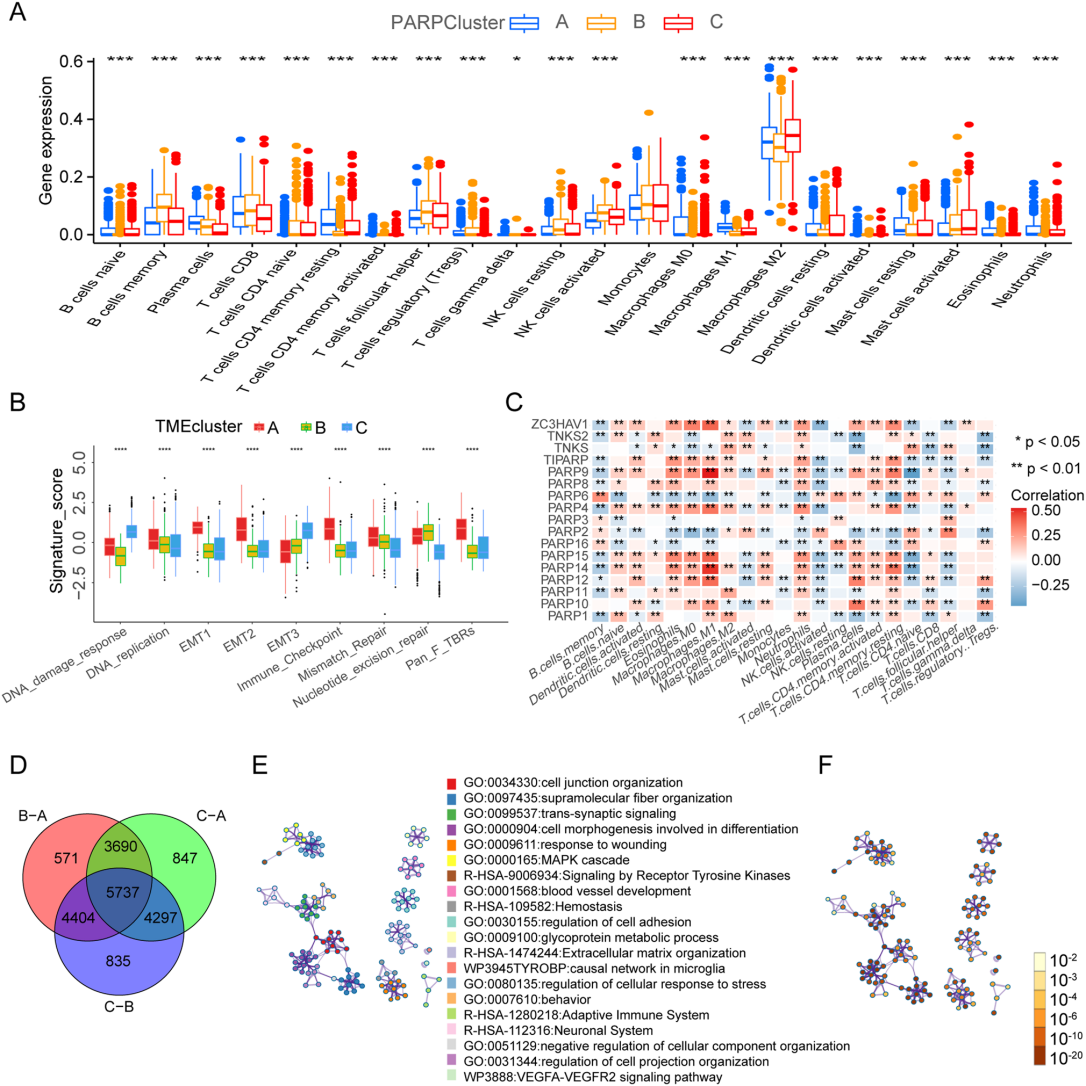
Association of PARPs Modification with TME Cell Infiltration. **A** Abundance of TME infiltrating cells in three PARPs modification patterns. **B** Differences in stroma-activated pathways, including EMT and TGF-beta pathways, among three distinct PARPs modification patterns. Statistical differences among the three modification patterns were tested using the one-way ANOVA test. **C** Correlation between each TME infiltration cell type and each PARP using Spearman analyses. Negative correlation marked with blue and positive correlation with red. **D** Venn diagram showing 5737 PARPs-related genes. **E** Functional annotation of the PARPs-related genes between three patterns in the combined glioma cohort. *p < 0.05; **p < 0.01; ***p < 0.001; ***p < 0.0001

We employed GSVA enrichment analysis to discern biological disparities among various PARPs modification patterns. Our findings demonstrated that PARP cluster-A exhibited enrichments in stromal and carcinogenic activation pathways. Conversely, PARP cluster-C displayed a notable association with immunosuppressive biological processes (Fig. 2E, F, and supplementary table S4). Further analysis using ssGSEA and TME revealed that cluster A comprised a higher abundance of natural killer cells, macrophages, eosinophils, and mast cells, indicating a strong correlation with matrix activation. Cluster B, in contrast, exhibited an immune-inflamed phenotype, distinguished by adaptive immune cell infiltration and pronounced immune activation. Meanwhile, PARP cluster C was identified as an immune-desert phenotype, characterized by significant immune suppression (Fig. 3A, B, and S3I). Previous research has elucidated the role of ADP‐ribosylation in the differentiation and functions of both innate and adaptive immune cells [33]. To investigate the role of PARPs within the TME, we performed Spearman's correlation analysis. The results revealed a positive correlation between the expression of genes such as ZC3HAV1, TIPARP, PARP4, PARP14, and PARP15 and the abundance of various immune cell types in gliomas (Fig. 3C). These results emphasize the connection between PARP expression and immune infiltrates.

### Generation of PARPs signatures and functional annotation

**Fig. 4:**
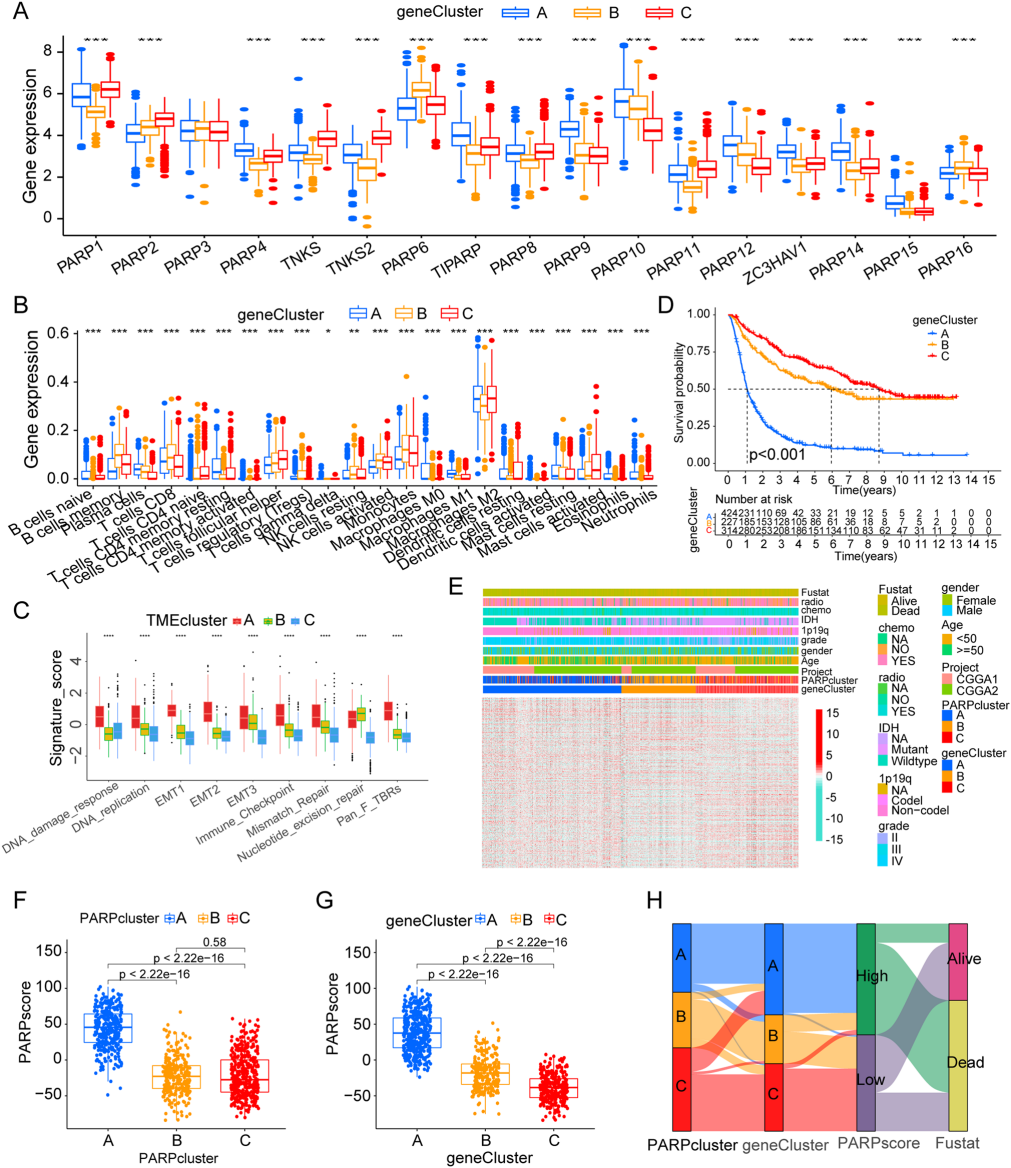
Construction of PARP Signatures. **A** Expression levels of 17 PARPs in three gene clusters. **B** Abundance of each TME infiltrating cell in three gene clusters. **C** Differences in stroma-activated pathways. **D** Survival analysis of glioma patients belonging to the three PARP-related gene clusters. **E** Heatmap illustrating consensus clustering based on PARPS-related differential genes. (F, G) Boxplots demonstrating the PARPscore for PARP patterns (**F**) and gene clusters (**G**). **H** Sankey diagram depicting the association between PARP patterns, gene clusters, PARPscore groups, and fustat. *p < 0.05; **p < 0.01; ***p < 0.001; ***p < 0.0001

We identified 5737 DEGs between different PARPs modification patterns using the “limma” package. The Venn diagram shows these results (Fig. 3D, supplementary table S5). GO enrichment analysis showed a significant correlation between biological processes such as PARPs modification and immune system regulation (Fig. 3E, F). To further screen for genes that have an impact on the overall survival of gliomas, we screened 4377 genes of the PARPs phenotype from 5737 DEGs by univariate cox regression and performed consensus clustering analysis based on these genes. Three subgroups were identified and named PARP gene cluster A-C (Fig. S4A-H, supplementary table S6). Unsurprisingly, the expression of PARPs in the 3 PARP gene clusters has obvious differences (Fig. 4A). We also repeated the above studies in the TCGA dataset and identified 3 gene clusters. And the survival analysis results between these three groups are also similar to those in the CGGA dataset (Fig. S5A).

### Clinical and relevant pathological features in PARP-associated phenotypes

**Fig. 5:**
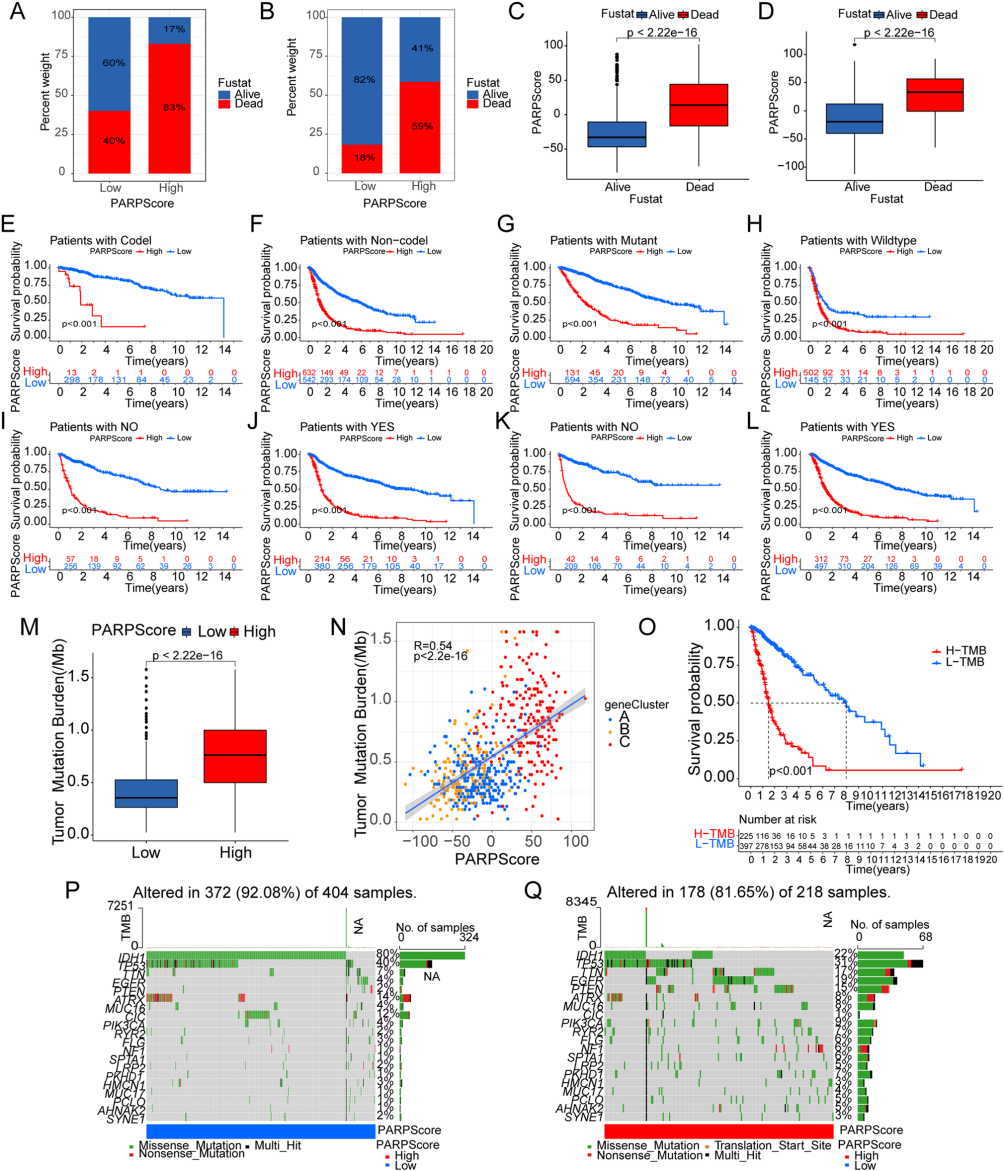
Relationship of PARP-modified Features to Tumor Somatic Mutations. **A**–**D** Differences in the proportion of overall PARPscore in different survival status groups of patients in CGGA (**A**, **C**) and TCGA (**B**, **D**). **E**–**L** Survival rates of patients with different 1p19q status (Codel and Non-codel; **E** and **F**), different IDH status (Mutant and Wildtype; G and H), with Radiotherapy or not (YES and NO; **I** and **J**), and with chemotherapy or not (YES and NO; **K** and **L**). **M** Differences in PARPscore between patients with high and low TMB. **N** Scatter plot depicting the relationship between PARPscore and TMB in glioma samples (R = 0.54; p < 2.2e−16). **O** The survival analyses of glioma samples in high and low TMB groups (p < 0.001). **P**–**Q** Waterfall plot presenting tumor somatic mutations in low (**P**) and high (**Q**) PARPscore subgroups

We conducted ssGSEA enrichment analysis and observed distinct enrichment patterns associated with immune activation, stromal activation, and carcinogenic pathways in PARP gene clusters C, B, and A, respectively. We validated these findings using consistent TME analysis methods (Fig. 4B and C). We also analyzed the differences in survival of these three gene clusters, and the results showed that the cluster C had the best prognosis (Fig. 4D). In addition, we used heatmap to display gene expression patterns between different clinical, and molecular phenotypes groups, and found clear differences in gene expression between each subgroup (Fig. 4E). Given the intricate nature of PARPs modification in gliomas, we introduced a scoring system termed PARPscore to evaluate PARPs modification patterns in glioma patients. PARPscore exhibited significant variations among different PARP gene clusters. Notably, PARP cluster A displayed the highest median score, while PARP clusters B and C showed no noticeable difference, potentially attributed to our dataset and PARP patterns. However, PARP gene cluster A exhibited the highest PARPscore, while cluster C had the lowest (Fig. 4F and G). The alluvial diagram effectively portrayed the attribute changes of individual patients (Fig. 4H). We stratified samples into high and low groups based on the PARPscore, revealing a significant survival advantage for patients with low PARPscore (p < 0.001, Fig. S4I). Univariate Cox regression (Fig. S5C and E) and multivariate Cox regression analyses (Fig. S5D and F), considering factors such as age, grade, IDH status, 1p19q status, etc., indicated that PARPscore is a valuable factor for assessing patient prognosis in both CGGA (Fig. S5C and D) and TCGA datasets (Fig. S5E and F). We further examined the differences in the overall PARPscore proportion among different survival status groups of patients. The analysis revealed higher overall PARPscore in the deceased group, whereas the PARPscore was lower in the surviving group (Fig. 5A, B, C, and D; A and C (CGGA), B and D (TCGA)). Additionally, we investigated the survival rates in patients with different 1p19q status (Codel and Non-codel; Fig. 5E and F), different IDH status (Mutant and Wildtype; Fig. 5G and H), with or without radiotherapy (YES and NO; Fig. 5I and J), and with or without chemotherapy (YES and NO; Fig. 5K and L). Overall, a high PARPscore was associated with the poorest survival rates. In the TCGA dataset, patients with low PARPscore also exhibited a significant survival benefit (p < 0.001, Fig. S5B).

### Relationship of PARPs-modified features to tumor somatic mutations

We utilized the "maftools" R package to analyze the distribution differences in somatic mutations between low and high PARPscore groups. Remarkably, we found that tumors with a low PARPscore were linked to reduced TMB (Fig. 5M and N). Notably, the low PARPscore group displayed a broader range of mutations compared to the high PARPscore group (Fig. 5P and Q). Additionally, our analysis indicated that patients with a combination of high PARPscore and high mutation burden experienced the poorest survival rates (Fig. 5O, Fig. S4J). These findings offer a novel perspective for investigating the relationship between PARP modification patterns and somatic mutations in tumors.

### The role of PARPs modification patterns in immunotherapy

**Fig. 6:**
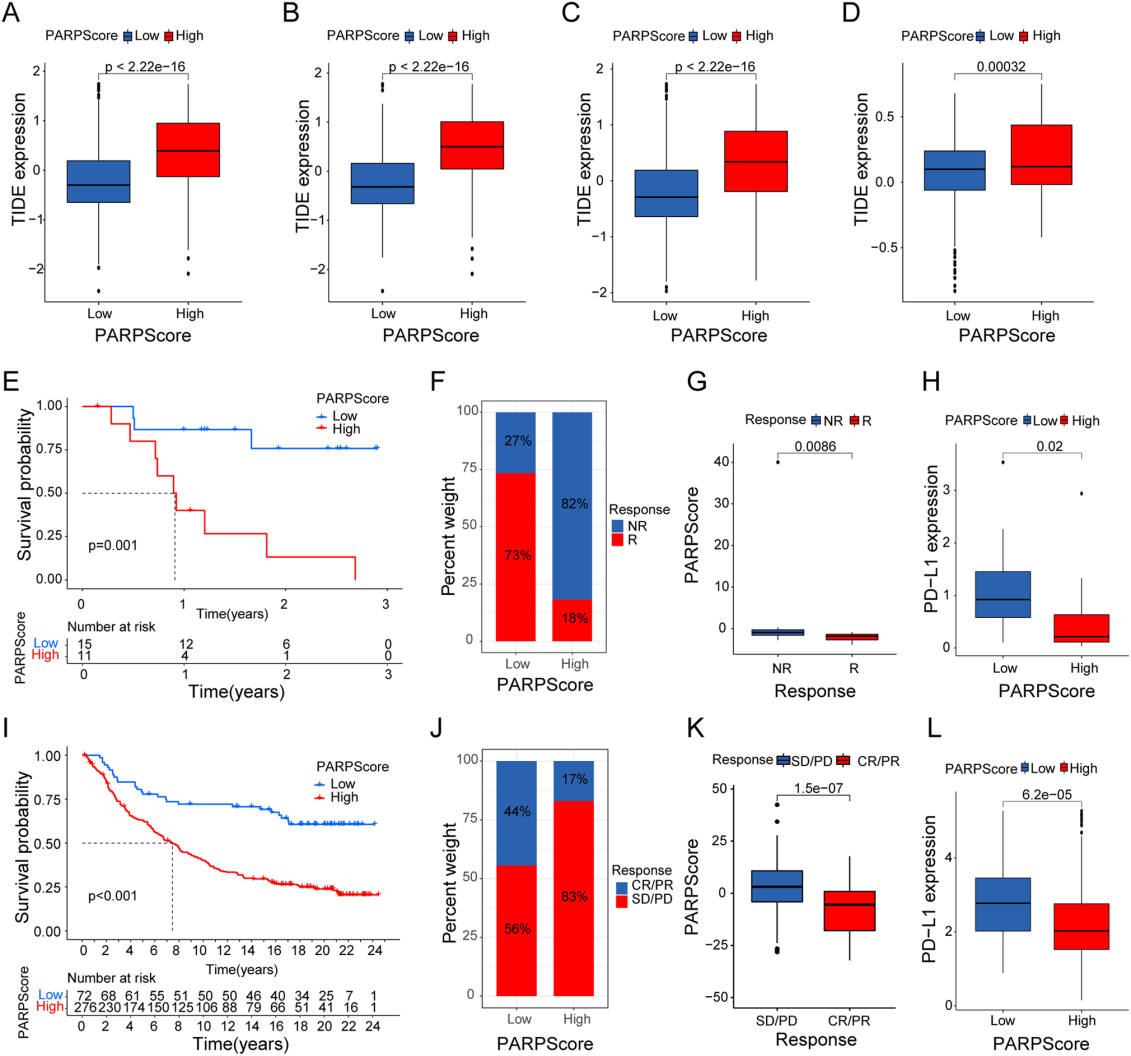
The Impact of PARP Modification Patterns on Immunotherapy. **A**–**D** Distribution of TIDE scores between high and low PARPscore subgroups in the gathered glioma cohorts (p < 2.22e−16) (**A**), as well as CGGA1 (p < 2.22e−16) (**B**), CGGA2 (p < 2.22e−16) (**C**), and TCGA (p = 0.00032) (**D**) datasets. **E**, **I** Survival analyses of OS for the low and high PARPscore subgroups in the two anti-PD-L1 immunotherapy cohorts (GSE79671, p = 0.001 (**E**) and IMvigor210, p < 0.001 (**I**)). **F**, **J** Proportion of patients responding to PD-1 blockade immunotherapy in the low and high PARPscore subgroups of the GSE79671 (**F**) and IMvigor210 (**J**) cohorts. **G**, **K** Clinical response to anti-PD-1 immunotherapy based on high or low PARPscore of patients in the GSE79671 (**G**, p = 0.0086) and IMvigor210 (**K**, p < 1.5e−07) cohorts. **H**, **L** Differences in PD-L1 expression between the PARPscore subgroups in the GSE79671 (**H**, p = 0.02) and IMvigor210 (**L**, p < 6.2e−05) cohorts

CTLA-4 and PD-1 blockade therapies have demonstrated significant improvements in survival rates across various cancer types [34, 35]. Building on the strong association observed between PARPscore and tumor immune response, we analyzed its correlation with TIDE scores. Our results revealed significantly lower TIDE scores in the high-PARPscore subgroup across the CGGA-merge, CGGA1, CGGA2, and TCGA cohorts (Fig. 6A–D). These findings highlight the substantial impact of PARPs on the tumor immune response in gliomas. To further examine the clinical relevance, we assessed the relationship between PARPscore and responses to anti-PD-L1/PD-1 immunotherapy in the IMvigor210 and GSE79671 cohorts. Patients with high PARPscore demonstrated poorer outcomes, with notably shorter survival times (GSE79671, p = 0.001; IMvigor210, p < 0.001, Fig. 6E and I). Patients in the high-PARPscore subgroup showed reduced responsiveness to PD-1/PD-L1 immunotherapy (Fig. 6F, G, J, and K). Furthermore, this subgroup exhibited significantly lower PD-L1 expression in both immunotherapy cohorts, providing insights into the potential molecular mechanisms influencing clinical immunotherapy responses (P < 0.05; Fig. 6H and L). The results demonstrate a significant association between PARP modification patterns and the immune response in gliomas. Furthermore, the PARPs signature accurately predicts clinical responses to anti-PD-1/PD-L1 immunotherapy.

## Discussion

Previous findings had suggested that PARPs modification is closely related to innate immunity and immune response [6, 36]. However, a comprehensive analysis of PARPs modification patterns and their effects on the immune microenvironment of gliomas remains to be studied. Therefore, study of the glioma immune microenvironment, as well as the identification of different PARPs modification patterns, will provide new insights into PARPs-based immunotherapy.

In our study, we identified three distinct PARP modification patterns based on the expression of PARPs using consensus clustering, and these patterns exhibited varying immune characteristics. Remarkably, these patterns aligned precisely with three classical immune phenotypes: immune inflammation, immune exclusion, and immune desert. Intriguingly, we observed that the immune inflammation phenotype was associated with the most favorable prognosis. This observation could be attributed to the fact that, although cluster A harbors a substantial number of immune cells, these cells are retained in the microenvironment surrounding the tumor without penetrating the tumor interior. The tumor microenvironment matrix may be confined to the tumor envelope or extend into the tumor, presenting an immune exclusion phenotype [37]. Hence, it is not surprising that the PARPs modification cluster A exhibited a worse prognosis than cluster C. ADP-ribosylation is implicated in inflammation and the regulation of adaptive immunity, including the regulation of T and B cell differentiation and activation [33]. In line with the above definitions, our analysis revealed that Cluster A displayed pronounced stromal activation, including elevated EMT, known for its immunosuppressive effects [29]. The TME cell-infiltration profiles across clusters further validated the robustness of our classification of immune phenotypes based on different PARPs modification patterns. Thus, it was unsurprising that Cluster A exhibited activated innate immunity yet was associated with a poorer prognosis, reflecting the distinct influence of PARPs modifications on TME characteristics. Previous studies have demonstrated that PARP1 deficiency leads to an increased number of Foxp3^+^ Treg cells in lymphatic organs [38], and PARP9 and PARP14 regulate macrophage polarization [39], these findings support the correlation of PARPs with immune cells and underscore the need for further investigation into the immune modification patterns of PARPs.

Poly (ADP-ribose) polymerase inhibitors (PARPi) and immune checkpoint inhibitors, including monoclonal antibodies targeting PD-1 and CTLA-4, have transformed cancer therapy [15]. Emerging evidence indicates that PARPi can influence the tumor's inflammatory immune microenvironment and restore a functional Th1 immune response. This dual immunological impact of PARPi has the potential to enhance the effectiveness of immune checkpoint inhibitors and strengthen antitumor immune responses [16]. Consequently, studying the role of PARP in immunotherapy is essential. Our study demonstrated that genes with differential expression across different PARPs modification patterns are significantly related to biological pathways associated with PARPs modification, such as tumor immunomodulation and immune response.

This study further revealed that variations in mRNA transcriptomes among distinct PARPs modification patterns were closely linked to PARPs- and immune-related biological pathways. The differentially expressed genes identified were designated as PARPs-related signature genes. Aligning with the clustering of PARPs modification phenotypes, three genomic subtypes emerged from these signature genes, demonstrating significant associations with stromal and immune activation. These findings highlight the pivotal role of PARPs modifications in shaping diverse TME landscapes.

To better understand TME cell infiltration patterns, it is essential to comprehensively assess individual PARPs modification patterns. Addressing this need, we developed and validated a scoring system, PARPscore, across multiple cohorts to quantify PARPs modifications. Tumors with an immune-excluded phenotype exhibited higher PARPscores, whereas those with an immune-inflamed phenotype showed lower scores. These findings were confirmed using the IMvigor210 cohort [29], where PARPscore differences aligned with immune phenotypes, suggesting its potential as a predictor of immunotherapy outcomes and a tool for tailoring glioma treatment strategies. Our findings underscore the critical role of PARPs modifications in immunomodulation and immune response, with significant implications for immunotherapy in gliomas. PARPs modification patterns were found to shape TME characteristics, notably the stromal and immune landscapes, which may impact the effectiveness of immune checkpoint blockade therapies. The PARPs gene signature, incorporating biomarkers like mutation burden, neoantigen load, PD-L1 expression, and TME attributes, presents a refined strategy for predicting immunotherapy outcomes. Furthermore, in two cohorts treated with anti-PD-1 and anti-PD-L1 therapies, PARPscore significantly differed between responders and non-responders, confirming its reliability as a robust indicator for personalized immunotherapy. These results underscore the non-negligible impact of PARPs modifications on TME dynamics and therapeutic efficacy, reinforcing their potential as a critical factor in advancing immunotherapy strategies for gliomas.

The PARPscore is a crucial clinical tool for assessing PARPs modification patterns and their related TME cell infiltration characteristics in individual patients, facilitating the identification of tumor immune phenotypes and guiding more effective clinical strategies. Additionally, the PARPscore can assess various clinicopathological features, including tumor inflammation stages, histological subtypes, and tumor mutation burden, as detailed in our study. It also functions as an independent prognostic biomarker for predicting patient survival. The PARPscore also proves valuable in predicting the effectiveness of adjuvant chemotherapy and clinical responses to anti-PD-1/PD-L1 immunotherapy. These findings provide a valuable foundation for improving immunotherapy outcomes, identifying distinct tumor immune phenotypes, and advancing personalized approaches in cancer treatment.

Our research has several limitations. First, obtaining a comprehensive immunotherapy dataset specifically for gliomas proved challenging. The immunotherapy validation cohort GSE79671 [30] we utilized comprised patients with recurrent glioma, while the IMvigor210 cohort consisted of advanced urothelial cancer samples. Although we observed significant differences between response and non-response groups, the disparity in tumor types may constrain the generalizability of our findings. Further extensive experiments are required to validate these results. Additionally, the role of the PARPscore in glioma treatment needs further confirmation in clinical settings. Second, differences in datasets across platforms may introduce minor biases into the study.

## Conclusions

In summary, our study delineates three distinct immune subtypes in gliomas based on the PARP perspective, and we developed a personalized PARP profiling scoring system, termed PARPscore. PARPscore proves to be a valuable tool for predicting survival outcomes, clinicopathological characteristics, and immunotherapy efficacy. This scoring system offers promising prospects for enhancing patients' clinical responses to immunotherapy, discerning diverse tumor immune phenotypes, and advancing the field of personalized cancer immunotherapy.

## Supplementary Information

Below is the link to the electronic supplementary material.

Supplementary file1 (DOCX 2289 KB)

Supplementary file2 (XLSX 164 KB)

## Data Availability

All data are available in a public, open access repository. The datasets used and/or analyzed during the current study are available in the Gene Expression Omnibus (GEO, https://www.ncbi.nlm.nih.gov/geo/), the Cancer Genome Atlas (TCGA) network (https://cancergenome.nih.gov/), and the Chinese Glioma Genome Atlas (CGGA, http://www.cgga.org.cn/). For other data generated during the analysis, please contact the corresponding author if necessary.

## Abbreviations

PARP: Poly (ADP-ribose) polymerase
TCGA: The Cancer Genome Atlas
CGGA: Chinese Glioma Genome Atlas
GEO: Gene Expression Omnibus
GBM: Glioblastoma
LGG: Low Grade Glioma
PCA: Principal component analysis
CNS: Central nervous system
ssGSEA: Single-Sample Gene Set Enrichment Analysis
GSVA: Gene set variation analysis
NBT: Normal brain tissue
TME: Tumor microenvironment
