# Supplementary material for: The PARPscore system using poly (ADP-ribose) polymerase (PARP) family features and tumor immune microenvironment in glioma
Source: Discov Oncol. 2024 Dec 26;15:839. doi: 10.1007/s12672-024-01734-2 (PMC11671460; doi:10.1007/s12672-024-01734-2)
Supplement: Supplementary file 1 — Supplementary file1 (DOCX 2289 KB) [file 12672_2024_1734_MOESM1_ESM.docx]

**Supplementary Figure**

**Supplementary Figure S1**

**
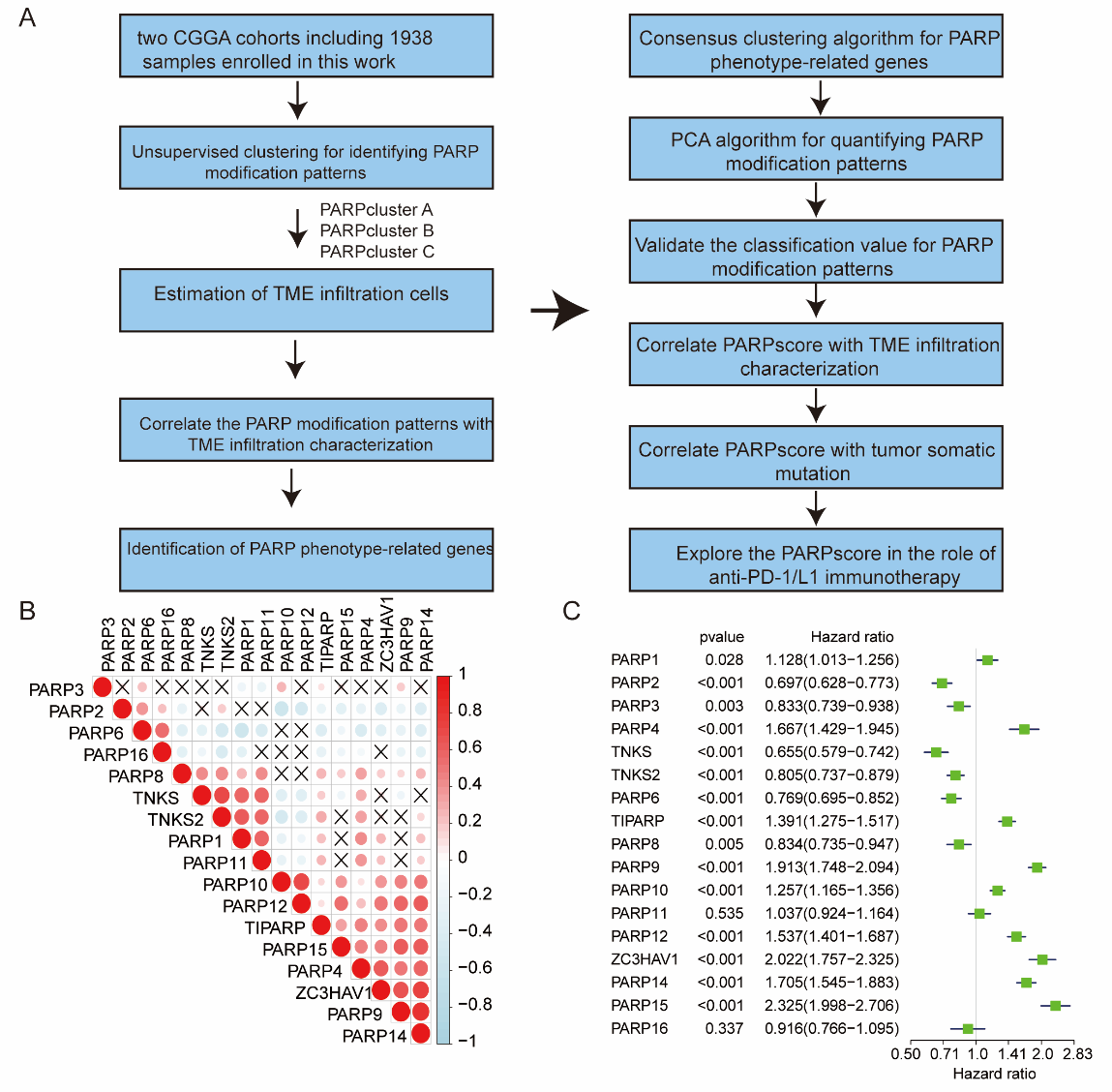
**

**Fig. S1 Overview of the design of 17 PARPs modification models.** (A) Overview of this work. (B) The mutation co-occurrence and exclusion analyses for 17 PARPs. Co-occurrencered; Exclusion, Blue. (C) The prognostic analyses for 17 PARPs in the CGGA cohorts using a univariate Cox regression model. Hazard ratio >1 represented risk factors for survival and hazard ratio <1 represented protective factors for survival.

**Supplementary Figure S2**


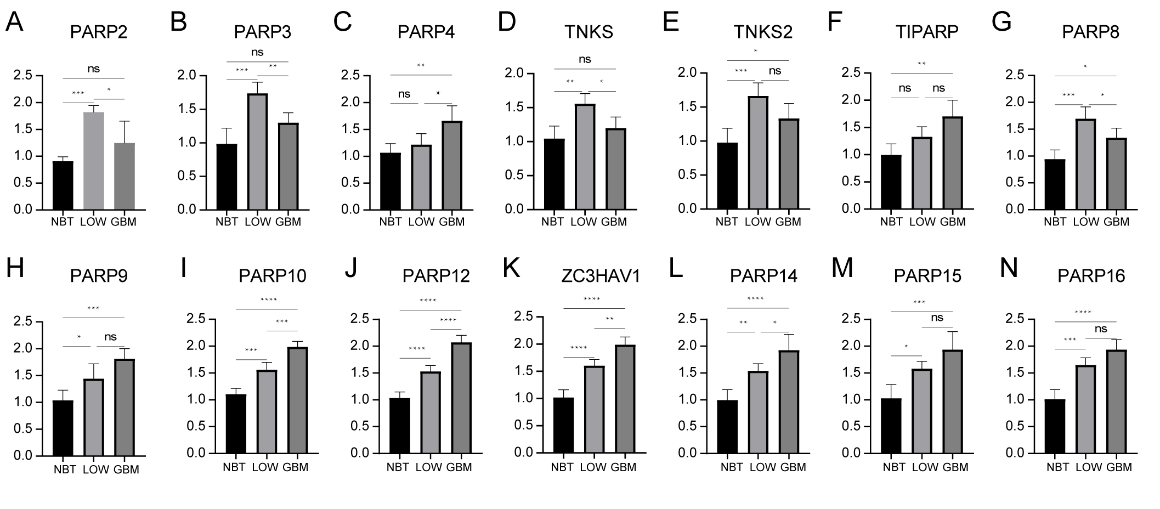


**Fig. S2 RNA expression levels of 17 PARPs.** (A-N) The mRNA expression patterns of 14 PARPs in non-neoplastic brain tissues and glioma tissues.

**Supplementary Figure S3**


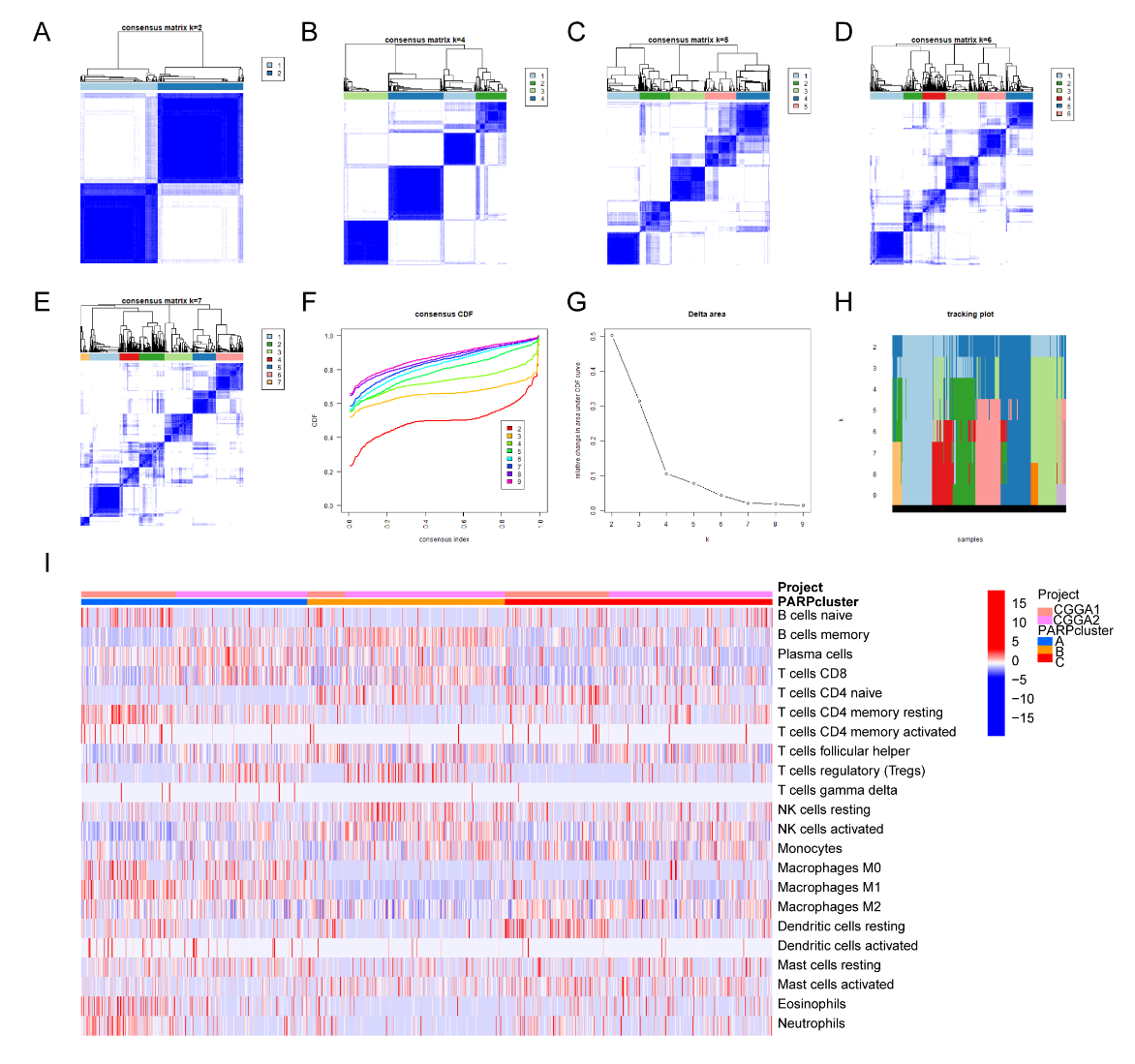


**Fig. S3** **Unsupervised clustering of 17 PARPs in glioma, dividing all patients into 3 clusters.** (A-E) Consensus matrices of the TCGA cohort for k = 2 - 6. (F) Consensus clustering cumulative distribution function (CDF) for k = 2-9.(G,H) Relative change in area under CDF curve for k =2-9. (l) Heatmap shows the GSVA score of representative Hallmark pathways in distinct PARPS modification patterns.

**Supplementary Figure S4**


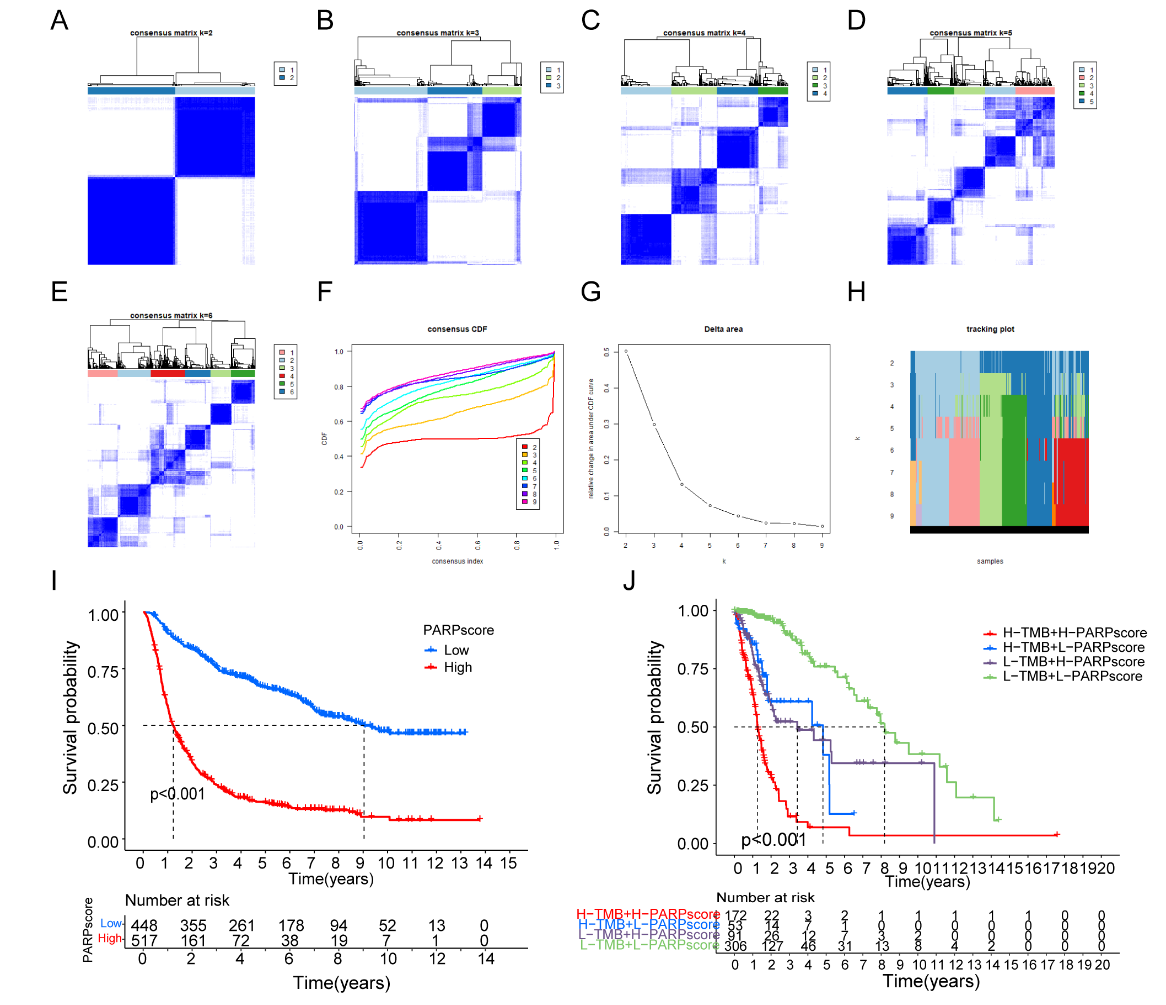


**Fig. S4 Using non-consensus clustering of gene transcription levels, all patient flowers were divided into 3 clusters**. (A-E) Consensus matrices of the TCGA cohort for k =2 - 6. (F) Consensus clustering cumulative distribution function (CDF) for k= 2-9. (G, H) Relative change in area under CDF curve for k = 2-9. (l) Survival analyses for low and high PARPscore patient groups in CGGA cohort using Kaplan-Meier curves. (J) Survival analysis of distinct groups stratified by both TMB and PARPscore.

**Supplementary Figure S5**


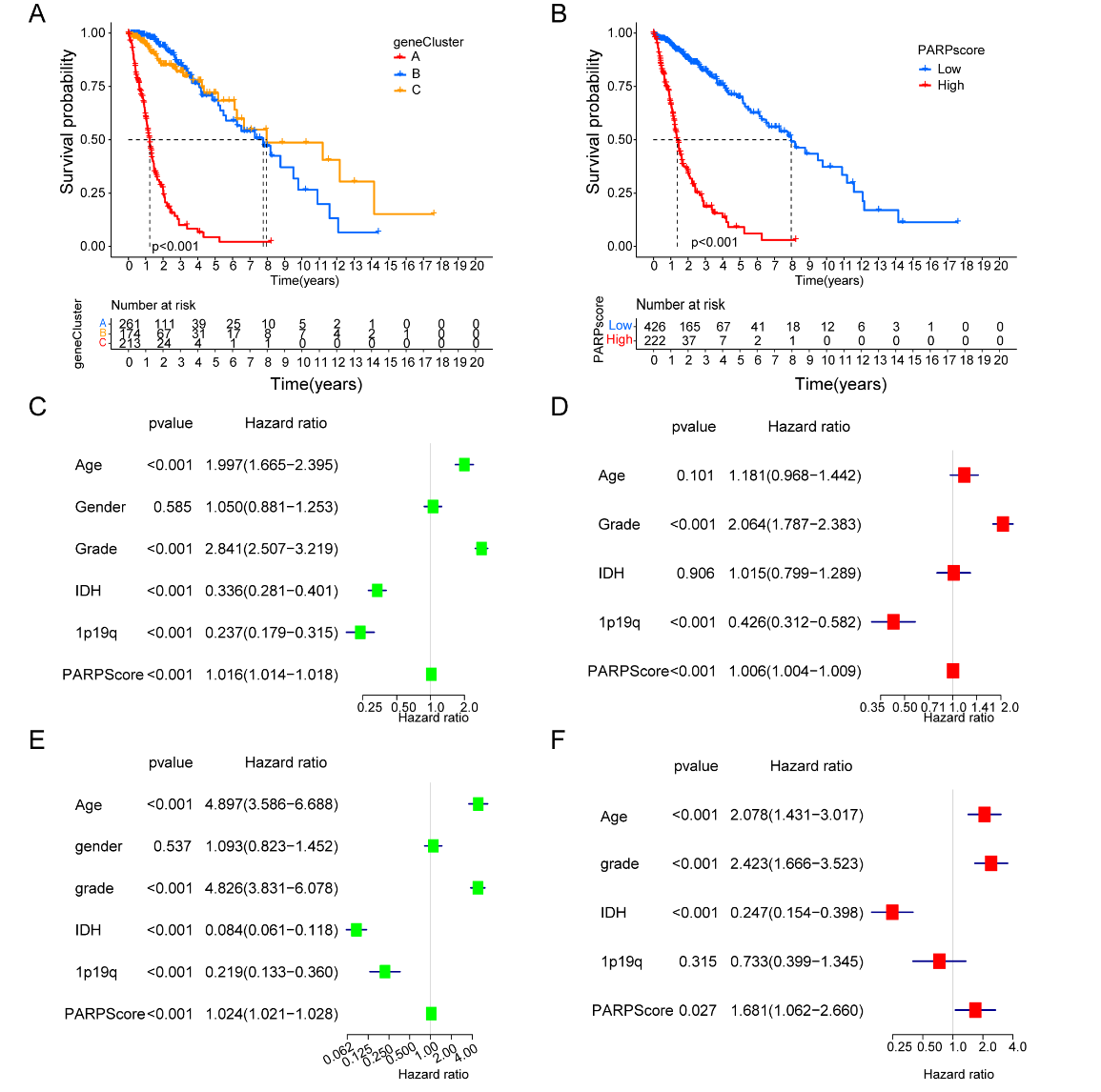


**Fig. S5 Univariate and multifactorial cox analyses for each clinical phenotype.** (A) Kaplan-Meier curves indicated PARP modification genomic phenotypes were markedly related to overall survival in TCGA cohort. (B) Survival analyses for low and high PARPscore patient groups in TCGA cohort using Kaplan-Meier curves. (C-F) Univariate Cox regression and Multivariate Cox regression analysis for PARPscore in CGGA cohort (C, D) and TCGA cohort (E, F) shown by the forest plot.
